# Clinical impacts of sarcopenic obesity on chronic obstructive pulmonary disease: a cross-sectional study

**DOI:** 10.1186/s12890-023-02702-2

**Published:** 2023-10-18

**Authors:** Zilin Wang, Xiaoming Zhou, Mingming Deng, Yan Yin, Yanxia Li, Qin Zhang, Yiding Bian, Jinrui Miao, Jiaye Li, Gang Hou

**Affiliations:** 1 National Center for Respiratory Medicine; State Key Laboratory of Respiratory Health and Multimorbidity; National Clinical Research Center for Respiratory Diseases; Institute of Respiratory Medicine, Chinese Academy of Medical Sciences; Department of Pulmonary and Critical Care Medicine, Center of Respiratory Medicine, China-Japan Friendship Hospital, Beijing, China; 2 Graduate School of Peking Union Medical College, Chinese Academy of Medical Sciences, Beijing, China; 3 Department of Pulmonary and Critical Care Medicine, First Hospital of China Medical University, Shenyang, China; 4 Respiratory Department, The First Affiliated Hospital of Dalian Medical University, Dalian, China; 5 Department of Pulmonary and Critical Care Medicine, Peking Union Medical College, Fuwai Hospital, Chinese Academy of Medical Sciences, Disease, Beijing, China

**Keywords:** COPD, Sarcopenic obesity, Sarcopenia, Resistin

## Abstract

**Background:**

Sarcopenia and obesity are two abnormal body composition phenotypes, and sarcopenic obesity (SO) is characterized by both low skeletal muscle mass (sarcopenia) and high adiposity (obesity). SO negatively influences the clinical status of patients with chronic obstructive pulmonary disease (COPD). However, the studies exploring the prevalence and clinical effects of SO in COPD patients are limited. Our study aimed to elucidate the prevalence and impact of SO on COPD patients.

**Methods:**

In this cross-sectional study, the pulmonary function, St. George’s Respiratory Questionnaire, exercise tolerance, body composition, and serum levels of resistin and TNF-α were assessed in 198 COPD patients. The clinical value of serum resistin and TNF-α for predicting SO in patients with COPD was evaluated.

**Results:**

In the 198 patients with COPD, the prevalence rates of sarcopenia, obesity, and SO in COPD patients were 27.27%, 29.8%, and 9.6%, respectively. Patients with SO experienced more severe symptoms of dyspnea and worse health related quality of life. The expression of resistin increased in patients with SO compared to other patients. The AUC value of serum resistin level for predicting SO was 0.870 (95% CI: 0.799–0.940). BMI (OR: 1.474, 95% CI: 1.124–1.934) and resistin (OR: 1.001, 95% CI: 1.000-1.002) levels were independent risk factors of SO in patients with COPD in Multivariate analysis.

**Conclusion:**

The prevalence rates of SO in COPD patients was 9.6%. COPD accompanied by SO is significantly associated with worse pulmonary function and poor physical performance. Serum resistin may be a potential adjunct for predicting SO in COPD patients.

## Background

Chronic obstructive pulmonary disease (COPD) is a heterogeneous lung condition characterized by airway and/or alveolar abnormalities that cause persistent, often progressive, airway obstruction [1]. COPD is a leading cause of mortality worldwide [2]. Significant extrapulmonary comorbidities in COPD patients, including diabetes, cardiovascular diseases, non-alcoholic fatty liver disease and sarcopenia, impact its clinical outcomes [3, 4]. Sarcopenia is characterized by skeletal muscle dysfunction and the loss of skeletal muscle mass [5]. COPD patients with sarcopenia have worse clinical outcomes, such as worse lung function and impaired exercise performance [4, 6, 7]. Notably, some COPD patients were observed to have other body composition abnormalities, including obesity [8]. Obesity is typically characterized by excess body fat accumulation [9]. COPD patients with obesity are considered to have a poorer health status and demonstrate increased comorbidities and mortality, impaired exercise performance, and decreased quality of life [8, 10, 11].

Sarcopenic obesity (SO) refers to the coexistence of sarcopenia and obesity [12]. COPD concurrent with SO presents a more complicated inflammatory response involving crosstalk between systemic inflammation and lipodystrophy that contributes to the occurrence and development of SO [13]. The abnormal expression of adipocytokines, such as resistin, plays a significant role in lipometabolism as well as in proinflammatory effects [14–16]. In addition, elevated levels of inflammatory factors are a critical manifestation of the systemic inflammatory response, in which elevated serum levels of tumor necrosis factor (TNF)-α are a risk factor for skeletal muscle dysfunction [17]. The role of resistin and TNF-α, as well as their interactions in COPD concurrent with SO, remains unclear.

However, studies exploring the effects of adipokines in patients with COPD stratified by obesity, sarcopenia, and SO are limited. Therefore, we conducted a cross-sectional study to explore the prevalence and impact of SO in COPD patients. Moreover, we evaluated the crucial roles of systemic inflammatory cytokines and adipokines in COPD patients with SO.

## Methods

### Study Design and participants

This was a cross-sectional study, approved by the Research Ethics Committee of the First Hospital of China Medical University (No. 2018-144-2). Participants consisted of patients with COPD were enrolled at the First Affiliated Hospital of China Medical University (Shenyang, China) from August 2018 to December 2019. Patients involved in this study were all adults (older than 18 years) and written informed consents were obtained from all patients. According to the Global Initiative for Chronic Obstructive Lung Disease (GOLD) criteria, patients who were diagnosed with stable COPD were enrolled. Patients meeting the following criteria were excluded: [1] the presence of any important comorbidities that would interfere with measurements of muscle function (e.g., uncontrolled diabetes, severe cardiovascular, neurologic, or orthopedic diseases); [2] history of acute exacerbations of COPD at least one month before this study; and [3] history of rehabilitation within one year.

### Data measurements

Spirometry measurements were recorded in the Jaeger MasterScreen system (Viasys Healthcare GmbH, Hochberg, Germany) following the American Thoracic Society and European Respiratory Society guidelines. The Chinese version of modified medical research council scale (mMRC) [18] was used to measure symptoms of dyspnea, and quality of life was measured using the COPD assessment test (CAT) [19]. St. George’s Respiratory Questionnaire (SGRQ) was used to assess health related quality of life (HRQoL). Exercise tolerance was evaluated by the 6-minute walking distance (6 MWD) in line with guidelines of the 2002 American Thoracic Society (ATS). Venous blood was collected in serum separator tubes while the patient was in a fasted state. The human resistin and TNF-α ELISA kits were used to detect resistin and TNF-α levels, per the manufacturer’s instructions (R&D, Minneapolis, MN, USA).

### Assessment of body composition phenotypes

Body composition was assessed by bioelectrical impedance analysis (BIA; InBody770, Seoul, Korea). Handgrip strength (HGS) assessed by a hand dynamometer (JAMAR Plus + Hand Dynamometer) was used to evaluate muscle strength. Physical performance was assessed using the five-time sit-to-stand test (5STS) based on a previous study [20]. The identification of sarcopenia referred to the Asian Working Group for Sarcopenia (AWGS) guideline [16] following the criteria: low muscle mass [bioelectrical impedance (M: <7.0 kg/m2, F: <5.7 kg/m2)] and low muscle strength [handgrip strength (M: <28 kg, F: <18 kg)] and/or poor physical performance (five-time chair stand test: ≥12 s). Obesity was defined as body mass index (BMI) ≥ 25.0 kg·m^− 2^ [21, 22]. Patients with co-existence of obesity and sarcopenia were diagnosed as SO [23]. Therefore, COPD patients were stratified into four subgroups according to the criteria above: sarcopenia, obesity, SO, and normal body composition.

### Statistical analyses

The Mann–Whitney U test (non-normal distribution) or t-test (normally distributed data) was used to compare differences in quantitative variables between groups. Pearson’s or Spearman’s correlation coefficient was calculated to determine the association between clinical variables and the serum level of resistin or TNF-α, depending on the distribution status of variables. Logistic regression was conducted to identify the corresponding risk factors of SO. Variables with a *P*-value < 0.05 in the univariate logistic analysis were included in the multivariate logistic regression. The clinical efficacy of serum resistin to predict SO in patients with COPD was evaluated by using the receiver operating characteristic curve (ROC) analysis. Variables are presented as mean values with standard deviations or percentages. A P-value < 0.05 was considered statistically significant in all analyses. SPSS software (version 13.0; IBM, Armonk, NY, USA) was used for all analyses.

## Results

### Baseline characteristics of patients with COPD

In total, 198 patients with COPD were enrolled in this study. As shown in Tables 1 and 33.33% were stratified as normal body composition, 29.8% were patients with obesity and without sarcopenia, 27.27% were those with sarcopenia only, and 9.6% were patients with SO. Detailed characteristics are shown in Table 1. Compared with other three groups, patients with SO were considerably older, and showed noticeably worse pulmonary function (FEV_1_, FEV_1_%predicted). They performed more impaired physical function (6MWD and 5STS) and markedly advanced GOLD stages. Finally, patients with SO presented noticeably worse body composition measurements such as BMI, body fat, skeletal muscle mass index (SMMI), and fat-free mass index (FFMI).

**Table 1:** Baseline characteristics of subjects

Variable	NBC (n = 66)	Obesity (n = 59)	Sarcopenia (n = 54)	Sarcopenic Obesity (n = 19)	*P*-value
Demographics					
Age, years	63.63 ± 8.78	64.85 ± 8.48	68.47 ± 6.68	71.27 ± 8.52^*^	0.012
Sex, m/f (%)	47/19	47/12	36/18	10/9	0.126
Pulmonary function					
FEV_1_, %predicted	1.78 ± 0.59	1.72 ± 0.65	1.31 ± 0.55^*, #^	1.29 ± 0.63^*, #^	0.002
FEV_1_/FVC, %	57.90 ± 8.55	56.61 ± 9.29	52.34 ± 11.84	54.21 ± 8.99	0.106
Physical function					
6MWD, m	410.2 ± 50.31	397.6 ± 68.70	322.4 ± 34.37^*, #^	267.7 ± 82.97^*, #, &^	< 0.001
5STS, s	6.43 ± 1.94	6.48 ± 1.29^*^	8.94 ± 2.21^*, #^	11.84 ± 2.20^*, #, &^	< 0.001
Body composition					
BMI, kg/m^2^	22.01 ± 2.21	27.92 ± 2.66	21.09 ± 3.15	27.17 ± 2.54	< 0.001
Body fat (%)	26.08 ± 6.28	31.77 ± 4.49^*, &^	27.19 ± 5.92^#^	34.95 ± 4.72^*, &^	< 0.001
FFMI (kg/m^2^)	16.88 ± 1.28	18.76 ± 1.63^*^	14.89 ± 2.23^*, &^	14.29 ± 2.50^*, &^	< 0.001
SMMI (kg/m^2^)	28.37 ± 3.52	26.75 ± 2.80	26.21 ± 2.81^*,^	23.90 ± 2.54^*, &^	< 0.001

NBC, Normal Body Composition

Values are given as percentage, mean (SD), or median (interquartile range) and χ^2^, 1-way ANOVA, or Kruskal–Wallis ANOVA on Ranks, P-values are indicated respectively, as appropriate

*P < 0.05 compared with normal body composition

#P < 0.05 compared with obesity

&P < 0.05 compared with sarcopenia

### Decreased symptoms of dyspnea and HRQoL (Health related quality of life) associated with sarcopenic obesity in patients with COPD

The relationship between symptoms of dyspnea, quality of life and SO in patients with COPD was analyzed (Table 2.). CAT and mMRC scores were generally used to evaluate the respiratory symptoms of dyspnea. In this study, patients with SO experienced considerably higher CAT and mMRC scores than other patients, which means more severe dyspnea. SGRQ is a multidimensional scoring system to evaluate HRQoL; in our research, patients with sarcopenic obesity exhibited considerably worse quality of life regarding higher SGRQ activity, impact, symptoms, and total scores than patients without sarcopenia. These results suggest that patients with sarcopenic obesity show more severe symptoms of dyspnea and worse HRQoL.

**Table 2:** Respiratory Symptoms and Health-Related Quality of Life Associated with Sarcopenic Obesity in Patients with COPD

Variable	NBC (n = 66)	Obesity (n = 59)	Sarcopenia (n = 54)	Sarcopenic Obesity (n = 19)	*P*-value
mMRC score	0.84 ± 0.99	1.59 ± 1.26^*^	1.90 ± 1.24^*^	3.09 ± 1.04^*, #, &^	< 0.001
CAT scores	10.69 ± 6.73	12.81 ± 7.18	16.26 ± 9.53^*^	23.30 ± 8.37^*, #, &^	< 0.001
SGRQ					
SGRQ activity score	25.47 ± 18.90	26.65 ± 23.46	36.35 ± 23.28	53.33 ± 18.04^*, #, &^	0.006
SGRQ impact score	10.61 ± 11.88	18.29 ± 17.41	25.64 ± 13.38^*^	66.40 ± 14.40^*, #, &^	< 0.001
SGRQ symptoms score	19.44 ± 13.70	26.65 ± 23.46	39.29 ± 21.78^*^	63.00 ± 14.74^*, #, &^	< 0.001
Total score	17.00 ± 13.86	25.29 ± 20.33	32.53 ± 16.47^*^	58.17 ± 14.11^*, #, &^	< 0.001

NBC, Normal Body Composition; mMRC, modified Medical Research Council dyspnea scale

Values are given as percentage, mean (SD), or median (interquartile range) and χ2, 1-way ANOVA, or Kruskal–Wallis ANOVA on Ranks P values are indicated respectively, as appropriate

*P < 0 0.05 compared with normal body composition

#P < 0.05 compared with obesity

&P < 0.05 compared with sarcopenia

### Clinical efficacy of serum resistin and TNF-α levels for predicting sarcopenic obesity in patients with COPD

**Fig. 1:**
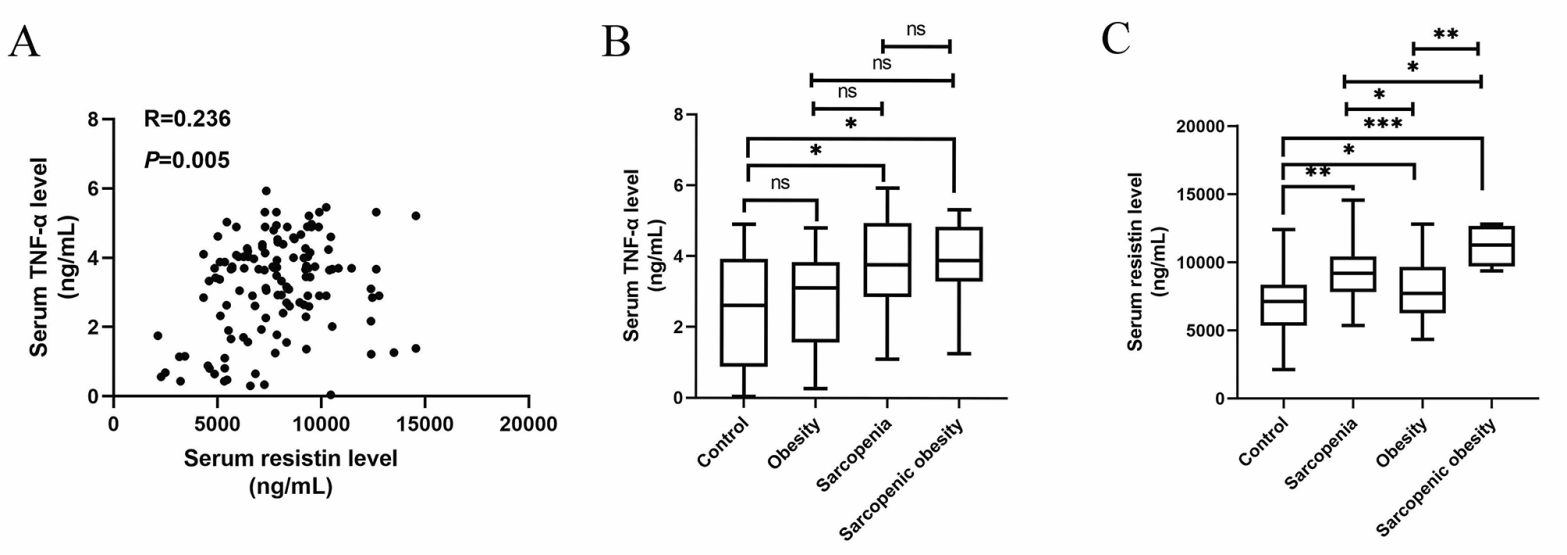
The clinical value of serum TNF-α and resistin level for predicting sarcopenic obesity. (**A**) The correlation between serum resistin and TNF-α level; (**B**) The serum TNF-α level is increased in patients with sarcopenic obesity compared to patients with normal body composition(control); (**C**) The serum resistin level is increased in patients with normal body composition(control), with sarcopenia, with obesity. ns, not significantly different; * P < 0.05, ** P < 0.01, *** P < 0.001

Systemic inflammatory factor (TNF-α) and resistin were indicated to be associated with sarcopenia and obesity in a previous study [24]. In this study, we verified the predictive efficacy of these parameters in patients with SO. Firstly, we analyzed the correlation between serum resistin and TNF-α level; the serum resistin levels were positively correlated with the serum levels of TNF-α (*r* = 0.236, *P* = 0.005) (Fig. 1A). Secondly, the expression of TNF-α and resistin increased in patients with SO compared to patients with normal body composition, with obesity and without sarcopenia, and with sarcopenia only (Fig. 1B and C).

**Fig. 2:**
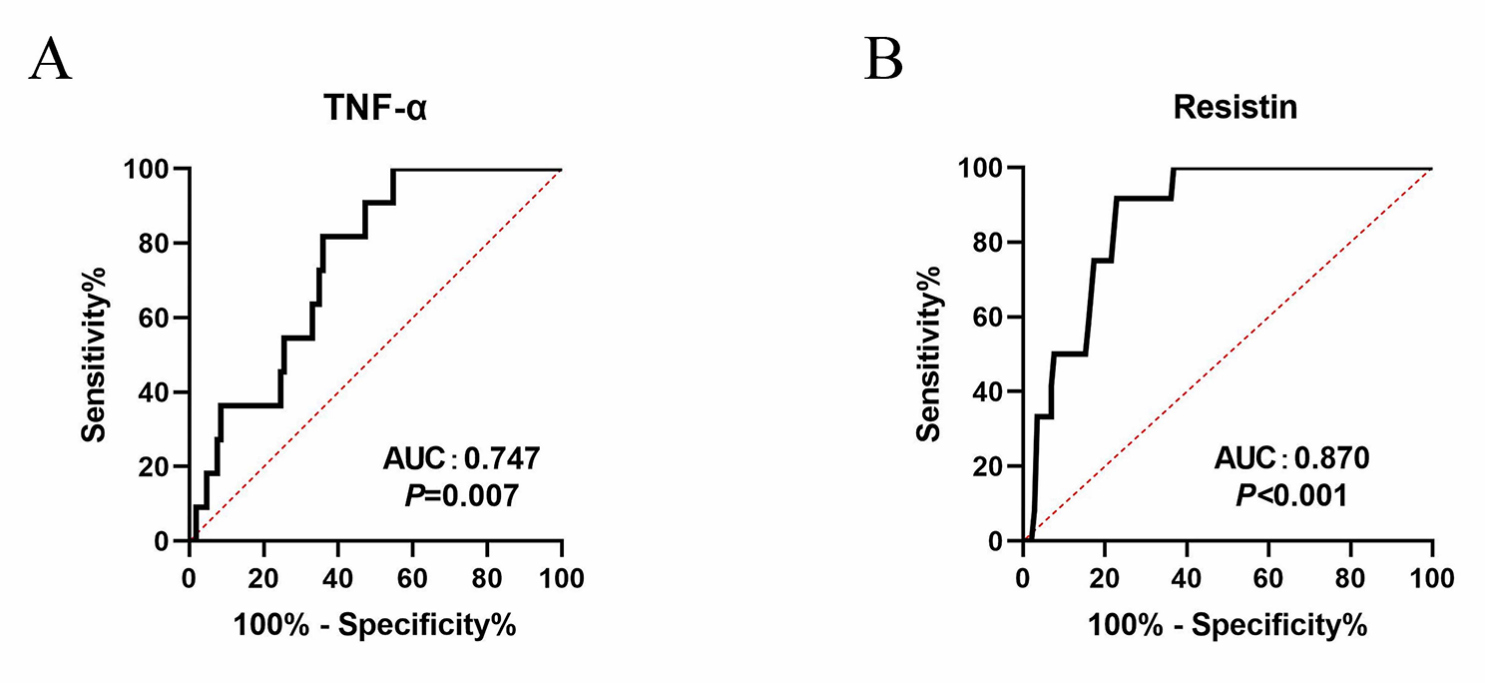
Receiver operating characteristic analysis of serum TNF-α and resistin level for predicting arcopenic obesity. (**A**) Receiver operating characteristic curve (ROC) curve of serum TNF-α level for sarcopenic obesity. (**B**) Receiver operating characteristic curve (ROC) curve of serum resistin level for sarcopenic obesity

In ROC analysis, the area under curve (AUC) value of serum resistin level for predicting SO was 0.870 (95% CI: 0.799–0.940), with a cutoff point of 9.342 ng/ml. The sensitivity and specificity of the serum resistin level were 91.67 and 77.08%. The serum level of TNF-α performed lower sensitivity (81.82%) and specificity (64.15%) to predict SO, with an AUC value of 0.747 (95% CI: 0.630 to 0.864, the cutoff point was 3.665 ng/ml). Overall, these results suggest that resistin may be associated with systemic inflammation, and serum resistin level may perform as an adjunct for predicting sarcopenic obesity in patients with COPD (Fig. 2A and B).

### The clinical factors associated with sarcopenic obesity

Univariate logistic analysis and multivariate logistic analysis were conducted to identify the potential clinical factors associated with SO (Table 3.). Age (OR: 1.107, 95% CI: 1.007–1.217, *P* = 0.036), BMI (OR: 1.284, 95% CI: 1.074–1.536, *P* = 0.006), serum resistin level (OR: 1.001, 95% CI: 1.000-1.001, *P* < 0.001), and serum TNF-α level (OR: 1.818, 95% CI: 1.041–3.177, *P* = 0.036) were associated with SO in patients with COPD in univariate analysis. Furthermore, we included age, BMI, serum resistin level, and serum TNF-α level into multivariate analysis, which showed that BMI (OR: 1.474, 95% CI: 1.124–1.934, *P* = 0.005), and serum resistin levels (OR: 1.001, 95% CI: 1.000-1.002, *P* = 0.001) were independent risk factors.

**Table 3:** Clinical factors associated with sarcopenic obesity in patients with COPD

Variable	Sarcopenia obesity				
	Univariate analysis			Multivariate analysis	
	OR (95%CI)	*P*-vale		OR (95%CI)	*P*-vale
Age	1.107(1.007–1.217)	0.036		1.106(0.960–1.274)	0.163
Sex	0.452(0.128–1.595)	0.217			
BMI	1.284(1.074–1.536)	0.006		1.474(1.124–1.934)	0.005
FEV_1_, %predicted	0.463(0.165–1.303)	0.145			
Serum Resistin	1.001(1.000-1.001)	< 0.001		1.001(1.000-1.002)	0.001
Serum TNF-α	1.818(1.041–3.177)	0.036		2.055(0.895–4.720)	0.089

## Discussion

In this study, we observed that 9.6% of COPD patients also had SO, and the prevalence was consistent with the Korean population but not the European population [25, 26]. This difference may be attributed to different racial genetics and dietary habits. The European population is more likely to have dyslipidemias [27–29], which contributes to fat redistribution and thus potentially causes SO [30]. This is the first cross-sectional study to disclose the nature of SO distribution in Chinese patients with COPD, thereby enriching the data on East Asian people.

We also observed a negative association between SO and exercise tolerance in COPD patients. When tested for exercise tolerance by the 6 MWT and 5STS, all patients with COPD stratified by body composition abnormalities (normal body composition, with obesity and without sarcopenia, and with sarcopenia only) exhibited impaired physical performance, whereas the performance was significantly worse for patients with SO. However, inconsistencies exist in the effects of obesity and COPD on exercise tolerance, as reported by *Ora et al.* [31]. and our results. We identified an association between obesity and reduced physical performance using the 6 MWT; however, the negative association disappeared when physical performance was evaluated using the cardiopulmonary incremental cycle test (CPET) [31, 32]. This was probably owing to the discrepant capability of the two tests in reflecting the mechanisms by which adipose tissues could impair physical performance [33]. CPET is a weight-supported exercise requiring mainly the legs to overcome externally imposed loads, whereas 6 MWT involves larger body segments. Patients with obesity, with excess fat mass and imbalanced contracting muscle, are required to overcome the extra load of their weight to perform the 6 MWT [34]. Therefore, the CPET has a limited ability to reflect the physical ability of patients with COPD and obesity compared with that of the 6 MWT. When tested using the 6 MWT, a similar reduction in exercise tolerance in obese patients was observed by *Joppa et al.* [25]. Further refinement of the evaluation method for exercise tolerance in patients with SO is required.

Oxidative stress in COPD and the “spillover” of lung inflammation into systemic circulation are the core pathophysiological mechanisms of extrapulmonary comorbidities, such as sarcopenia and metabolic abnormalities like obesity [3, 35]. The crosstalk between systemic inflammatory factors and adipokines might play a crucial role in the development of SO [36], comprising the process of muscle protein degradation and skeletal muscle ectopic fat infiltration [13]. TNF-α contributes to these processes by acting as a systemic inflammatory factor. Mechanistically, it can act on the skeletal muscle and lead to muscle atrophy by mediating muscle apoptosis by directly acting on TNF receptor 1 (TNFR1) [37]. The activation of TNFR1 suppresses AMP-activated protein kinase (AMPK) activity and consequently causes intramuscular lipid accumulation, leading to lipotoxicity-mediated insulin resistance [38], all of which comprise the myocellular biological pathways of SO [39]. The finding that patients with SO have higher levels of TNF-α in circulation than those with concurrent obesity or sarcopenia is consistent with the potential mechanism in the present and previous studies [25, 26]. Nevertheless, in addition to pulmonary and metabolic diseases, the multiple roles of TNF-α in an array of pathophysiologies, including cancer, neurological diseases, and cardiovascular diseases [40], which might influence the predictive value of TNF-α in SO, cannot be ignored.

Resistin, a member of the adipokine family, is secreted by macrophages in humans [41]. It also mediates insulin resistance via impaired insulin PI3K-mTOR signaling [14, 42], which downregulates protein synthesis in skeletal muscles [42]. Insulin resistance may also be related to the decreased myogenic differentiation of myoblasts by resistin [43]. Additionally, resistin interacts with TNF-α by upregulating its expression via NF-κB [44]. In contrast, several inflammatory cytokines, including TNF-α, can induce resistin expression in various cells [15]. Resistin and TNF-α play vital roles in lipotoxicity, muscle insulin resistance, and mitochondrial dysfunction, resulting in the pathophysiology of the development of SO in patients with COPD [13]. Our results provide evidence demonstrating a significantly higher serum level of resistin in patients with COPD concomitant with sarcopenia and obesity compared with that in patients with concurrent obesity or sarcopenia only. Furthermore, the serum resistin level was more closely associated with SO than TNF-α in patients with COPD, with a better AUC in terms of the predictive efficacy. This may be partly explained by the involvement of resistin in the modulation of lipids in skeletal muscle mass and function [14, 16, 39].

To date, sarcopenia definiation has published and updated by the European Working Group on Sarcopenia in Older People and the Asian Working Group for Sarcopenia perspectively, with the concern of different body size, cultural or lifestyle between European and Asian population. Given the Asian participants in our study, the definition of sarcopenia in our present study followed the guidelines from the Asian Working Group for Sarcopenia 2019 (AWGS). Moreover, uniform diagnostic criteria for SO have not been established, and researchers in a majority of studies currently combine the diagnostic criteria of sarcopenia with BMI or BFR to define SO [25, 26, 45]. However, patients could gain fat and lose muscle mass without changes in BMI, and FFMI also fails to reflect fat redistribution. Thus, it is necessary to explore several indicators that reflect more than just the status of muscle loss and fat gain. A serum biomarker panel with a combination of systemic inflammatory cytokines and adipokines might be an adjunct for predicting SO in patients with COPD.

### Limitation

First, the population in our study could only partly reflect the status of SO in Chinese patients with COPD; the reprensentative prevalence of SO in Chinese patients with SO may need a national cross-sectional study or meta-analysis. Second, BIA is not the uniformed tool for evaluating body composition abnormality. Though it was widely used in previous studies owing to its easy accessibility, which made it comparable between different population, BIA fails to evaluate adipose distribution, which might also be a potential factor contributing to exercise tolerance. Third, we could not identify cause–effect relationships between these proinflammatory cytokines and the progress of SO in patients with COPD owing to the cross-sectional nature of the study. More cohort studies with larger sample sizes and long-term follow-ups are warranted to provide comprehensive insight into SO in patients with COPD.

## Conclusion

In conclusion, we observed that patients with COPD and concurrent SO tended to have worse pulmonary function, more severe symptoms of dyspnea, and impaired exercise tolerance. Serum resistin levels might be used as an adjunct to predict SO in patients with COPD.

## Data Availability

The data presented in this study are available on request from the corresponding author. The data are not publicly available due to ethical reason.

## Abbreviations

SO: sarcopenic obesity
COPD: chronic obstructive pulmonary disease
TNF-α: tumor necrosis factor (TNF)-α
mMRC: modified medical research council
GOLD: Global Initiative for Chronic Obstructive Lung Disease
CAT: COPD assessment test
SGRQ: St. George’s Respiratory Questionnaire
ATS: American Thoracic Society
HRQoL: health related quality of life
6 MWD: 6-minute walking distance
BIA: bioelectrical impedance analysis
HGS: Handgrip strength
5STS: five time sit-to-stand test
BMI: body mass index
ROC: receiver operating characteristic curve
AWGS: Asian Working Group for Sarcopenia
FFMI: fat-free mass index
SMMI: skeletal muscle mass index
AUC: area under curve
CPET: cardiopulmonary incremental cycle test
TNFR1: TNF receptor 1
